# Delineation between different components of chronic pain using dimension reduction – an ASL fMRI study in hand osteoarthritis

**DOI:** 10.1002/ejp.1212

**Published:** 2018-04-16

**Authors:** D. Keszthelyi, Q. Aziz, J.K. Ruffle, O. O'Daly, D. Sanders, K. Krause, S.C.R. Williams, M.A. Howard

**Affiliations:** ^1^ Division of Gastroenterology Department of Internal Medicine Maastricht University Medical Center Maastricht the Netherlands; ^2^ Centre for Neuroscience and Trauma Blizard Institute Wingate Institute of Neurogastroenterology Barts and the London School of Medicine and Dentistry Queen Mary University of London UK; ^3^ Department of Neuroimaging Institute of Psychiatry, Psychology & Neuroscience at King's College London UK; ^4^ Pain Management Research Institute The University of Sydney at Royal North Shore Hospital St Leonards NSW Australia; ^5^ Department of Neurology Philipps‐University Marburg Marburg Germany

## Abstract

**Background:**

Traditional psychometric measures aimed at characterizing the pain experience often show considerable overlap, due to interlinked affective and modulatory processes under central nervous system control. Neuroimaging studies have been employed to investigate this complexity of pain processing, in an attempt to provide a quantifiable, adjunctive description of pain perception. In this exploratory study, we examine psychometric and neuroimaging data from 38 patients with painful osteoarthritis of the carpometacarpal joint. We had two aims: first, to utilize principal component analysis (PCA) as a dimension reduction strategy across multiple self‐reported endpoints of pain, cognitive and affective functioning; second, to investigate the relationship between identified dimensions and regional cerebral blood flow (rCBF) as an indirect measure of brain activity underpinning their ongoing pain experiences.

**Methods:**

Psychometric data were collected using validated questionnaires. Quantitative estimates of rCBF were acquired using pseudo‐continuous arterial spin‐labelled functional magnetic resonance imaging.

**Results:**

Two principal components were identified that accounted for 73% of data variance; one related to pain scores and a second to psychological traits. Voxel‐wise multiple regression analysis revealed a significant negative association between the ‘pain score’ component and rCBF to a right temporal lobe cluster, including the amygdala and the parahippocampal cortex.

**Conclusion:**

We suggest this association may represent a coping mechanism that aims to reduce fear‐related pain‐anxiety. Further investigation of central brain processing mechanisms in osteoarthritis‐related pain may offer insights into more effective therapeutic strategies.

**Significance:**

This study demonstrates that dimension reduction using PCA allows insight into pain perception and its affective components in relation to brain activation patterns in patients with painful hand osteoarthritis.

## 1 Introduction

Management of chronic pain is often challenging, primarily due to its complex nature, but also due to the lack of reliable instruments for quantifying an individual's experience of pain. Common approaches rely on various self‐report questionnaires, each designed to capture specific aspects of the pain experience (Younger et al., 2009). As not one instrument currently appears to assess all features of chronic pain, multiple questionnaires are often used to provide a more comprehensive description of chronic pain perception, each of which are thought to reflect different constructs. Although these various constructs are considered conceptually separate, there often is considerable overlap of information, thus making their interpretation difficult.

More recently, noninvasive neuroimaging studies have been employed to investigate the complex brain regions involved in pain processing, in an attempt to provide a quantifiable, adjunctive description of pain perception. Previous brain imaging studies have mainly focused on investigating responses to painful evoked stimuli using blood oxygenation level‐dependent (BOLD) functional magnetic resonance imaging (fMRI), a technique best suited to recording responses transient to repeated stimuli with a time course of less than one minute. By contrast, the arterial spin labelling (ASL) fMRI modality has increasingly been applied in the field of pain research to investigate brain activity relating to longer duration background or spontaneous pain, a feature of many chronic pain states (Owen et al., 2008, 2010). ASL provides quantitative, reproducible whole‐brain measures of regional cerebral blood flow (rCBF) that are strongly associated with neuronal activity (Attwell et al., 2010; Howard et al., 2011). For example, a recent study of capsaicin‐induced heat pain in healthy volunteers, which identified distinct neural correlates to pain sensation (Segerdahl et al., 2015).

It is increasingly acknowledged that neuroimaging studies investigating pain should take into account psychological factors such as anxiety and depression as they are often associated with chronic pain states (Jamison and Edwards, 2012). Various studies have attempted to understand the relationship between self‐reported estimates, psychological factors and brain imaging (Hsu et al., 2009; Apkarian, 2010; Ivo et al., 2013). Such approaches may be limited by widely acknowledged covariance in psychometric parameters. Furthermore, multiple testing of each psychometric measure with brain imaging data may result in false‐positive results (Vul et al., 2009).

In this study, we examine psychometric and fMRI data from a group of patients with spontaneous pain secondary to osteoarthritis (OA) of the carpometacarpal (CMC) joint of the right thumb. Common to other chronic pain states, OA pain has a significant affective component (Siviero et al., 2016). OA of the thumb CMC joint has a reported radiographic prevalence of 7% in men and 15% in women (Haara et al., 2004). Many patients remain minimally symptomatic; however, a subset of patients develop debilitating pain, weakness and instability that severely limit hand function. Pain in OA is traditionally believed to originate from increased peripheral discharge as a result of degenerative and remodelling processes in the joints and synovia. However, current evidence suggests that, even though OA joint damage predisposes to pain, little correlation between pain severity and the extent of joint damage exists, suggesting central modulatory effects on nociceptive input (Kidd, 2012). Recent neuroimaging studies in OA patients have indeed highlighted a significant central nervous system (CNS) component in maintaining ongoing pain characteristic of the disease (Gwilym et al., 2009; Howard et al., 2012; Thakur et al., 2014; Sanders et al., 2015).

The aim of this exploratory study was to apply dimension reduction to pain scores and an array of psychometric data in CMC OA patients, with the intention of identifying a reduced number of principal determinants of these data. We then examined the relationship between these components and the brain representation of their spontaneous pain, as indexed by rCBF. As we did not have prior hypotheses about the anatomical location of these relationships, we adopted a hypothesis‐free whole‐brain voxel‐by‐voxel multiple linear regression analysis approach.

## 2 Methods

### 2.1 Data source and ethical approval

Data were originated from two previously acquired studies (Howard et al., 2012; Sanders et al., 2015). One study compared CMC OA patients to matched controls (study A), the second sought to determine the analgesic effects of naproxen versus placebo in two separate sessions (clinicaltrials.gov registration number NCT00830050; study B). In the latter study, ASL data from placebo only sessions were included in the current analysis. Ethical approval was obtained from The Joint South London and Maudsley and The Institute of Psychiatry NHS Research Ethics Committee (reference no 07/H0807/69 and 10/H0807/10, respectively). Written informed consent was obtained from all participants.

### 2.2 Study participants

Postmenopausal female participants were recruited from multiple sources, including national radio, local and national print magazines, a university e‐mail circular and referrals from hospital rheumatology and physiotherapy departments. All had a confirmed diagnosis of OA of the first CMC joint of the right hand according to American College of Rheumatology criteria (Altman et al., 1990) and were right‐hand dominant with a background pain intensity score of their affected limb of 3 or more on an 11‐point (0–10) numerical rating scale (NRS) at screening or randomization assessments. All participants had a disease duration of at least 1 year and maintained a stable drug regimen during study participation. Analgesic use other than NSAIDs, paracetamol or compound medications of NSAIDS/paracetamol with low‐dose codeine was prohibited for a minimum of one week prior to the scanning visit, to ensure no confounding effects on brain or psychometric questionnaire data. Normal exclusion criteria for MRI applied, including extremes of height and weight, the presence of internal metal and claustrophobia. Several other exclusion criteria were also applied, including severe pain elsewhere in the body that might impair or confound the assessment of OA‐related pain, as well as a history of other severe acute or chronic medical or psychiatric conditions.

### 2.3 Screening and familiarization

At the initial session, all patients underwent a comprehensive medical and blood screening, which also include an assessment of medication history. A clinical hand examination was performed by a specialist physiotherapist. Urine drug screen and alcohol breath tests were administered prior to MRI scanning to ensure no confounding drug or alcohol use. Prior to acquiring MRI data, each participant underwent a ‘mock scan’ in a simulated MRI environment, designed to reduce feelings of anxiety and claustrophobia.

### 2.4 Psychometric assessment

All participants completed psychometric, personality trait and pain‐related questionnaires prior to MRI acquisition. The short form McGill Pain Questionnaire (MPQ) was completed by all participants, which contains 15 words describing pain (11 sensory, four affective), 1 item on present pain intensity (PPI) and 1 item visual analogue scale (VAS) for pain (Melzack, 1987). The Beck Depression Inventory‐II (BDI) assesses depression (BDI range 0–21), each answer being scored on a scale value of 0 to 3 (Beck et al., 1996). A total BDI score above 14 indicates mild depression, moderate above 20 and severe over 28. The Spielberger State‐Trait Anxiety Inventory (STAI) was used for assessment of trait anxiety (STAI range, 20 – 80; higher scores represent higher anxiety–Spielberger et al., 1983). In addition, participants completed the revised Eysenck Personality Questionnaire (EPQR) (Eysenck, 1991). For the purpose of this study, only neuroticism levels (EPQR‐N) were assessed (neuroticism range, 0–24; higher scores represent higher neuroticism). The Patient‐Rated Wrist and Hand Evaluation (PRWHE) was also completed (MacDermid and Tottenham, 2004). The PRWHE is a 15‐item (five pain and 10 disability items) questionnaire that rates pain and disability of the hand/wrist in functional activities. Scoring is achieved by summing the five pain items and the 10 disability items, and dividing by 2. Scores range from 0 to 100, with 0 reflecting no pain/disability.

### 2.5 MRI data acquisition

MRI data were acquired on a 3T Signa HDx whole‐body scanner (General Electric) fitted with an 8‐channel, phased‐array receive‐only head coil. Assessments of regional CBF throughout the entire brain were acquired using a pseudo‐continuous arterial spin‐labelled (pCASL) perfusion MRI sequence. Continuous ASL sequences provide superior labelling efficiency compared to pulsed ASL techniques, resulting in an improved signal‐to‐noise ratio. The pseudo‐continuous ASL variant is adopted by virtue of its compatibility with modern body coil transmission hardware now ubiquitous on clinical MRI scanners. Accordingly, pCASL is the ASL variant of choice that is increasingly recommended for clinical imaging (Alsop et al., 2015).

Images were acquired at a 48 × 64 × 60 matrix on an 18 × 24 × 18 cm field of view. Images were reconstructed to a 256^2^ matrix. Sixty slices of 3 mm thickness were obtained, resulting in an actual voxel size of 3.75*3.75*3 mm. A full technical description of all pCASL parameters including rCBF computation is located at http://www.kcl.ac.uk/ioppn/depts/neuroimaging/research/pain/pCASLdetail.pdf.

In a single scanning session, two pCASL scans were collected with a duration of 6 min and spaced 20 min apart. Between scans, a motor task was performed in both studies, described in Sanders et al., (2015). Participants performed an isometric squeeze task designed to transiently evoke pain. The task was slow‐paced with very short‐duration stimulation periods (approximately one second), with the intention of avoiding any sensitization effects over the course of the task. The two pCASL scans were averaged as a means of improving the signal‐to‐noise ratio, as demonstrated in a previous study (O'Muircheartaigh et al., 2015). A high‐resolution T1‐weighted three‐dimensional (3D) spoiled gradient‐recalled acquisition in the steady state (SPGR) sequence was also acquired for interparticipant registration.

### 2.6 Statistical analyses

#### 2.6.1 Behavioural data analysis

Pain scores and psychometric data were analysed using SPSS, version 20 (Armonk, NY). Descriptive statistics of all scores and a correlation matrix for all variables were generated using Pearson's correlation coefficients. The threshold for statistical significance was chosen at a value of *p *<* *0.05. In order to examine the distribution of variances among variables and to perform dimension reduction, eight variables including PRWHE, STAI, BDI, MPQ (sensory, affective, VAS and PPI) and EPQR‐N were included in a principal component analysis (PCA) (Tabachnick, 2007). First, Kaiser–Meyer–Olkin measure of sampling adequacy and Bartlett's test of sphericity were performed. The former is a statistic test that indicates the proportion of variance in variables that might be caused by underlying factors. High values (close to 1.0) generally indicate that a factor analysis is suitable for the data test. The latter tests the hypothesis that the correlation matrix is an identity matrix, which would indicate that variables are unrelated and therefore unsuitable for structure detection. Small values (<0.05) of the significance level indicate that a factor analysis is suitable for the data set.

PCA itself is a mathematical procedure that applies orthogonal transformation to a number of correlated variables into a smaller number of uncorrelated variables called principal components. Orthogonal transformation determines several orthogonal lines of best fit to the data set. The greatest variance of the data set comes to lie on the first axis (then called the principal component). Each succeeding component accounts for as much of the remaining variability as possible under the constraint that it is orthogonal to the preceding components. The amount of variation in the total sample accounted for by each factor is expressed as an eigenvalue. The correlation between the component and the original variables is called the component loading. Following generation of these principal components, additional rotation of the factor axes is applied to determine a more simplified and interpretable pattern. Oblique (direct oblimin) rotation was used as this technique relaxes the orthogonality constraint and allows components to be correlated. In addition, we performed the PCA analysis using an alternative method of rotation (varimax), a type of rotation method that is strictly orthogonal. Hereafter, the principal components extracted with an eigenvalue of >1 (according to the Kaiser criterion) were used as explanatory variables in a univariate analysis of ASL data (see below).

#### 2.6.2 Functional MRI data processing

Image preprocessing was conducted using Statistical Parametric Mapping software (SPM8) (http://www.fil.ion.ucl.ac.uk/spm/software/spm8/) and the ASAP ASL toolbox (Mato Abad et al., 2016). Preprocessing included reorientation, within‐subject co‐registration of the 3D SPGR structural image to the ASL images, skull‐stripping, segmentation, normalization to Montreal Neurological Institute (MNI152) template space and spatial smoothing using a full‐width half‐maximum Gaussian kernel of 8 mm. An average CBF map derived from the two ASL volumes was computed for each participant. A measure of global CBF was computed for each participant, defined as the mean of all grey matter voxels.

#### 2.6.3 MRI data analysis

Multiple regression analysis by a general linear model was performed in SPM using each of the two factors derived from the PCA separately as regressors of interest. Patient age and global CBF values were included as covariates of no interest. We report clusters surviving family‐wise error correction on the basis of spatial extent at *p *<* *0.05, according to the Gaussian random field theory (Worsley et al., 1992). In addition, rCBF values of the suprathreshold clusters were extracted using MarsBaR toolbox for SPM and entered into SPSS to perform partial linear regression analysis for plotting and to detect any outliers.

## 3 Results

A total of 47 patients were included. MRI images from four patients were excluded due to insufficient image quality due to image artefacts, claustrophobia and MRI hardware failure. Demographic data were incomplete for one patient. Therefore, data from 42 patients were extracted for analysis. All brain images were assessed for anomalies by a trained neuroradiologist. One patient had severe cerebral atrophy and hence was excluded. Three patients were excluded as the brain images did not include the cerebellum. Therefore, data from 38 patients were included in the final analysis set (20 from study A, 18 from study B; see Table 1).

**Table 1:** Descriptive statistics of demographic characteristics and psychometric scores

	Total population	Study A (20 patients)	Study B (18 patients)	*p* value
Age (years)	60 (38–76)	60.5 (38–76)	60 (52–72)	0.76
BDI	4 (0–26)	4 (0–25)	4 (0–26)	0.41
PRWHE	27 (7–87)	27 (7–87)	26 (14–73)	0.46
STAI	46 (25–80)	45 (25–67)	46 (36–80)	0.61
EPQR‐N	9 (1–20)	9 (1–20)	9 (1–20)	0.72
MPQ‐sensory	6 (0–23)	5 (0–14)	6 (3–23)	0.08
MPQ‐affective	0 (0–7)	0 (0–3)	0 (0–7)	0.48
MPQ‐VAS	25 (0–82)	17 (0–69)	31 (8–82)	0.02
MPQ‐PPI	1 (0–4)	1 (0–4)	1 (1–2)	0.30

Data indicated as median (range). *p* values relate to Mann–Whitney *U*‐test, not corrected for multiple testing. BDI, Beck depression inventory; PRWHE, patient‐rated wrist and hand evaluation; MPQ, McGill pain questionnaire (11 sensory, four affective items), one item on present pain intensity (PPI) and 1‐item visual analogue scale (VAS); STAI, Spielberger state‐trait anxiety inventory; EPQR‐N, revised Eysenck personality questionnaire, neuroticism levels.

### 3.1 Psychometric data analysis

Descriptive summary statistics of the psychometric data are provided in Table 1. Overall, patients represent a clinically nondepressed population. No significant differences were observed in psychometric data between the two sources of the current patient cohort apart from McGill VAS (*p* = 0.02, uncorrected for multiple testing; Table 1).

Interrelationships between the different variables, assessed using Pearson's correlation coefficients, are provided in Table 2. Psychometric scores BDI, STAI and EPQR‐N were significantly correlated. In addition, there was a significant positive correlation found between the pain scores measured by the McGill and PRWHE questionnaires. EPQR‐N scores were weakly but significantly correlated with PRWHE and affective MPQ scores. PRWHE scores showed a positive correlation with BDI scores, but not with STAI scores. BDI scores correlated with all other variables and PRWHE also correlated with all but one (STAI).

**Table 2:** Correlation matrix of variables

	BDI	PRWHE	STAI	EPQR‐N	McGill sensory	McGill affective	McGill VAS
PRWHE	0.384**						
STAI	0.644***	0.081					
EPQR‐N	0.746***	0.369**	0.614***				
McGill sensory	0.329*	0.643***	0.097	0.182			
McGill affective	0.398*	0.604***	0.122	0.270*	0.669		
McGill VAS	0.296*	0.766***	0.093	0.227	0.706***	0.618	
McGill PPI	0.270*	0.659***	0.134	0.227	0.397**	0.374**	0.612***

Values indicate correlation coefficients according to Pearson. BDI, Beck depression inventory; PRWHE, patient‐rated wrist and hand evaluation; MPQ, McGill pain questionnaire (11 sensory, four affective items), one item on present pain intensity (PPI) and 1‐item visual analogue scale (VAS); STAI, Spielberger state‐trait anxiety inventory; EPQR‐N, revised Eysenck personality questionnaire, neuroticism levels.

*p <* *0.05, **p < 0.01, ***p < 0.001.

### 3.2 Dimension reduction

**Figure 1:**
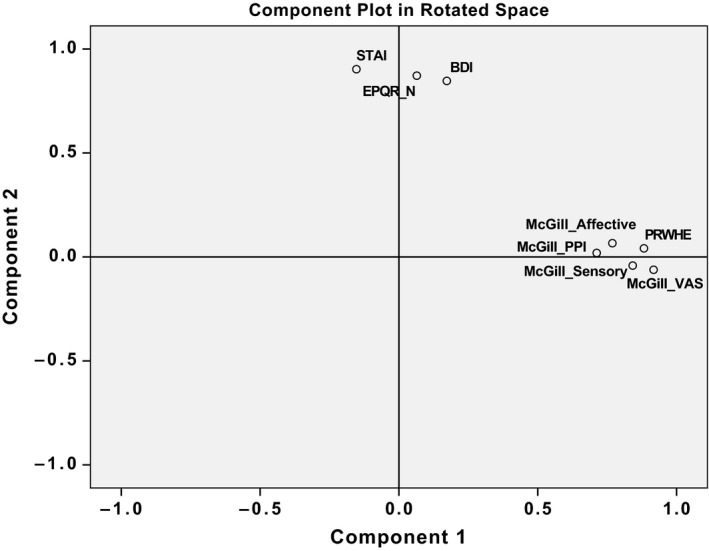
Principal component rotation plot of the variables. BDI, Beck depression inventory; PRWHE, patient‐rated wrist and hand evaluation; MPQ, McGill pain questionnaire (11 sensory, four affective items), one item on present pain intensity (PPI) and 1‐item visual analogue scale (VAS); STAI, Spielberger state‐trait anxiety inventory; EPQR‐N, revised Eysenck personality questionnaire, neuroticism levels.

Kaiser–Meyer–Olkin measure (value 0.8) and Bartlett's test of sphericity (*p* < 0.0001) both indicated that the data set was suitable to perform factor analysis. We performed dimension reduction using PCA and oblique rotation, identifying two principal components that accounted for 73% of the total variance. Component 1 (accounting for 49.7% of the variance, eigenvalue 3.98) was highly related to the pain scores PRWHE and all elements of the McGill questionnaire (see Fig. 1 for two‐dimensional rotation plot of principal components and variables and loading scores in Table 3). This indicates similar sources of variance for PRWHE and McGill scores. The second principal component accounted for 23.2% of the variance (eigenvalue 1.85) and principally related to the psychological traits (STAI, BDI and neuroticism). Performing PCA with strictly orthogonal (varimax) rotation revealed similar results (data not shown).

**Table 3:** Loading scores (component coefficients) for variables and components as derived from principal component analysis

Variables	Component 1	Component 2
McGill sensory	0.830	0.040
McGill affective	0.778	0.139
McGill VAS	0.901	0.027
McGill PPI	0.713	0.087
STAI	0.021	0.871
BDI	0.334	0.847
PRWHE	0.886	0.125
EPQR‐N	0.231	0.862

A loading score represents the strength of the correlation between a certain variable to the principal component (range 0–1.0). BDI, Beck depression inventory; PRWHE, patient‐rated wrist and hand evaluation; MPQ, McGill pain questionnaire (11 sensory, four affective items), one item on present pain intensity (PPI) and 1‐item visual analogue scale (VAS); STAI, Spielberger state‐trait anxiety inventory; EPQR‐N, revised Eysenck personality questionnaire, neuroticism levels.

### 3.3 Voxel‐wise multiple regression analysis

**Figure 2:**
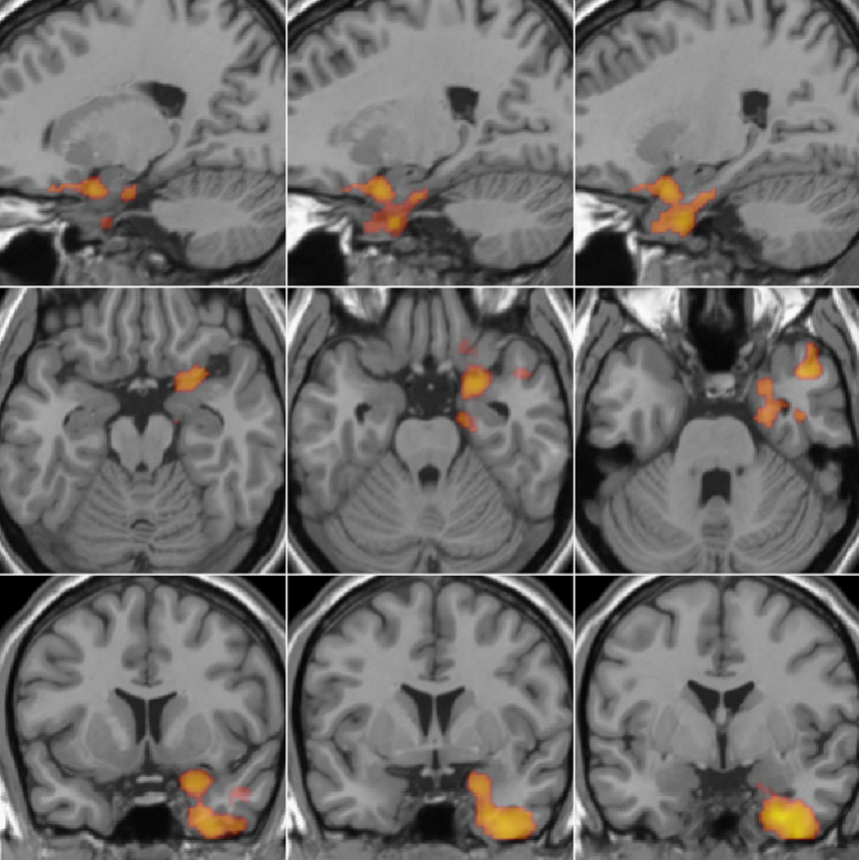
Brain region showing a negative association with Component 1 as derived from principal component analysis of psychometric data. The large cluster in the right hemisphere included the amygdala and piriform cortex, parahippocampal–entorhinal, fusiform and inferior temporal gyri, extending into the temporal pole and adjacent orbitofrontal cortex.

Multiple regression analysis, using the principal components derived from the PCA and fMRI ASL‐derived rCBF data, showed a significant negative relationship with Component 1 (‘pain scores’ component) in a large cluster (*k* = 1400, *p *=* *0.028) in the right hemisphere, which included the amygdala and piriform cortex, parahippocampal–entorhinal, fusiform and inferior temporal gyri, extending into the temporal pole and adjacent orbitofrontal cortex (see Fig. 2).

Regional CBF values were extracted for the large cluster described above and visualized in Fig. 2. Partial linear correlation was executed with these values correcting for global CBF (see Supporting Information Fig. S1) showing no evidence of outliers. We did not identify any significant relationship between rCBF and Component 2.

## 4 Discussion

In this study of patients with persistent pain secondary to OA of the CMC joint of the hand, we performed an exploratory analysis PCA using dimension reduction across a wide range of pain and psychometric scores. We identified two principal components responsible for almost 75% of the variation of data – one relating primarily to pain scores and the second to psychological trait (trait anxiety, neuroticism, depression). We examined the relationship of these components to resting‐state rCBF and found a negative association between the regional blood flow of a large area in the right hemisphere and the principal component related to pain scores but not the component related to psychological trait.

It is increasingly accepted that in chronic pain, emotional and psychological factors become more important than peripheral input itself in pain perception, as the relation between peripheral input and subjective pain experience is highly nonlinear (Baliki and Apkarian, 2015). Clinical evidence suggests that the perception of chronic pain can be maintained even though the peripheral signs of the injury, and thus a source of nociceptive activity, have decreased in intensity or even disappeared (Apkarian, 2010). Therefore, identifying emotional‐affective pain neuromodulatory mechanisms located in the central nervous system have important clinical implications, as analgesic therapies often do not prove effective in chronic pain states (Borsook et al., 2014). In an attempt to delineate between pain perception and psychological influences, including depression and trait anxiety, we performed PCA, with the intention that this would give a more distinct indication of the interrelationships between psychometric variables, before then seeking to understand their relationship with patterns of brain activity reflecting each patient's ongoing pain experience. This method also reduces the number of tests needed to describe this relationship, obviating any further multiple comparison correction for repeated assessments. We identified two principal components related to pain scores and psychological trait, respectively. These results are in line with findings on pain scores with those from Mounce et al., (2010), who identified separate principal components in a large sample from the healthy population using nine different psychometric questionnaires: one related to pain and another to depression, anxiety and negative affectivity. It is of note, however, that the second component identified in our study seems to be composed of measures of underlying psychological traits (e.g. trait anxiety), but as this does not incorporate any state measurements, the other dimension, which relates to pain scores, may incorporate affective components of this nature, such as state anxiety and pain expectation.

Following PCA analysis, we examined the relationship of these extracted components to rCBF, a surrogate marker for brain activation. Given the common clinical observation that chronic pain conditions are comorbid with depression and anxiety (Rubin, 2007; Tunks et al., 2008) and that anticipating and being anxious about pain can exacerbate the pain experienced (Ploghaus et al., 2001), we had expected that rCBF would be affected by increases in depression and/or anxiety, such as recently described in painful knee OA using ASL (Cottam et al., 2016). We interpret our findings on the lack of association between psychological trait and rCBF in the light of the findings of Hashmi et al., (2013)*,* describing a study in patients with chronic back pain. In this study, a transitional shift in brain activity was seen in acute/subacute to chronic back pain from the acute pain circuit to the emotion‐related circuitry including the amygdala, suggesting that the brain's perception of pain is transformed from pain‐oriented to a focus on the specific emotions of pain as a part of pain chronification. In this regard, the pain percept is thought to reflect less its sensory properties related to peripheral nociceptive drive, but instead becoming a heightened and more complex emotional state, constructed from learning and resultant memory traces of the presence and persistence of the condition rather than related to the peripheral input *per se*. Interestingly, this transitional shift was *independent* of psychological factors, including depression and anxiety. Hashmi et al., (2013) suggest that the properties that capture the qualitative subjective salience of the pain seem sufficient to shift its representation from acute pain to emotion circuitry, irrespective of psychological comorbidities.

Consistent with the findings of Hashmi et al.*,* highlighting the superior role of the emotional circuitry in chronic pain, we found that important constituents of this circuitry – including the amygdala, entorhinal cortex and parahippocampal gyrus – have a significant relation to the ‘pain score’ component derived from our study population of hand OA patients. These areas are involved in affective aspects of pain perception, anticipatory anxiety and associated learning and have been shown to be activated in experimental settings involving painful stimuli (Liotti et al., 2000; Petrovic et al., 2000; Vachon‐Presseau et al., 2013). We observed, however, a *negative* relationship between the ‘pain scores’ component and rCBF. We hypothesize that the decreased blood flow over this area in relation to pain could represent decreased activity of a network involved in fear‐related anxiety of pain. In other words, we speculate on the basis of the current findings that a feature of patients with chronic painful hand OA have altered brain activity in the limbic cortex, possibly an attempt to suppress negative affective memories related to their ongoing pain experience. Previous studies have suggested that such deactivation can be part of a cognitive coping mechanism for pain suppression (Hsieh et al., 1999; Petrovic and Ingvar, 2002). Typically, this occurs when the subject is unable to make a behavioural response to avoid an aversive context or painful event (Napadow et al., 2007). Another potential explanation to the observed negative relationship between rCBF and pain scores could be related to increased blood flow reflecting better activation of inhibitory modulatory systems in the midbrain/brain stem, which are less effective in chronic pain. In the current analyses, however, we did not observe any significant rCBF changes in these brain areas.

Anecdotal and clinical evidence suggests that patients with osteoarthritis of the hand avoid using their affected joint as patients are aware that this would result in pain. Joint pain associated with OA has a strong mechanical component, triggered by specific movements or actions and relieved by rest (Felson, 2011). The question therefore arises whether such behavioural adaptation (avoiding the use of the affective joint) is related to decreased peripheral nociceptive input due to immobilization or that this reflects the hedonic learning memory of avoidance as imprinted in specific brain circuits. The latter explanation seems likely as alteration in brain circuitry in chronic pain is believed to be the reflection of both the suffering and coping strategies that impinge on learning and memory and on hedonics of everyday experience (Apkarian, 2010).

In order to ascertain the complex interaction of peripheral drive (including both noxious and innocuous input) and an altered brain circuitry in chronic pain, we recommend the collection of surrogate markers of the peripheral component is warranted in addition to brain imaging. Future studies of chronic osteoarthritic pain should therefore evaluate surrogate markers of peripheral discharge or nerve sensitization – such as nerve physiology studies, measurement of local inflammatory mediators (Schaible et al., 2011), which we were not present in the current data examined here. It is to be noted further that this study investigated a specific patient group of hand OA patients. It is therefore difficult to establish whether our findings can be generalized for chronic pain states as the brain activity signature for different chronic pain conditions may also be distinct. Changes observed here may very well be specific for this patient population. For instance, avoiding the use of an affected joint in hip or knee OA is more difficult in everyday functioning and these conditions may therefore be characterized by different ‘chronic pain neurosignatures’ (Baliki et al., 2011; Cottam et al., 2016).

Our exploratory analysis employed a relatively uncommon approach in brain imaging studies using PCA for dimension reduction to examine the putative relation between perceived ongoing pain intensity and psychological trait with regard to rCBF. We used the pCASL technique that has increasingly been recognized as a tool for investigation of ongoing pain states. We here theorize the existence of a potential coping strategy in chronic pain related to hand OA. We assume on the basis of our current findings using dimension reduction that identifying and manipulating processes underlying the emotional suffering (cortical–limbic circuitry) may be more successful in treating chronic pain in hand OA patients. However, this finding should be interpreted by caution due to a number of limitations. Our sample size was relatively small, and therefore, findings are of an exploratory nature and should be reproduced in larger populations. We only examined female patients to exclude for gender confounds. In addition, we did not have information on current or past drug use or on the exact duration of disease, although all patients had their condition for at least 12 months. These data have potential benefit as additional explanatory variables and collecting these data should be considered in future studies. We acknowledge a potential caveat in our study design. The data in the current study were derived from two separate patient cohorts, one of which from the placebo session of a drug study. Given that placebo effects in pain are acknowledged, this could have potentially influenced our results. On the other hand, no significant differences were demonstrated between the two patient cohorts in terms of demographics or psychometric scores, apart from the McGill VAS score (*p* = 0.02, uncorrected for multiple testing).

We used the PCA method in order to coalesce multiple questionnaire variables, so as to further probe the nature of pain‐related psychometric data. The method can offer slightly different outcomes, since following extraction there is an infinite number of rotations available and the results might therefore depend on the choice for factor extraction and calculation, although the two different rotation methods used here revealed similar results. Nevertheless, this method allows more insight into data characteristics and assists interpretation of analyses related to psychometric scores. Therefore, the PCA dimension reduction method arguably warrants further substantiation in pain studies.

In conclusion, we attempted to delineate between the different components of pain perception using dimension reduction by PCA in patients with painful OA of the CMC joint of the thumb. We showed a negative association with brain activation in a large area including the amygdala related to pain scores. This may represent a coping mechanism that aims to reduce fear‐related pain‐anxiety. Even though pain indices highly correlated with psychological characteristics, the alterations in brain activation were seen independently of psychological trait. Based on these results, we believe that further investigation of central brain processing mechanisms in OA‐related pain may offer insights into more effective therapeutic regimes.

## Author contributions

All authors were involved in drafting the article or revising it critically for important intellectual content, and all authors approved the final version to be published. Dr. Howard and Dr. Keszthelyi had full access to all of the data in the study and take responsibility for the integrity of the data and the accuracy of the data analysis. Keszthelyi, Aziz, Williams and Howard involved in the study conception and design. Sanders, Krause and Howard involved in the acquisition of data. Keszthelyi, Aziz, Ruffle, O'Daly, Williams and Howard analysed and interpreted the data.

## Supporting information


**Figure S1** Partial regression plot showing regional blood flow values extracted from the cluster found to be associated with component 1 derived from the principal component analysis.
