# Supplementary figures and images for: Delineation between different components of chronic pain using dimension reduction – an ASL fMRI study in hand osteoarthritis
Source: Eur J Pain. 2018 Apr 16;22(7):1245–54. doi: 10.1002/ejp.1212 (PMC6055802; doi:10.1002/ejp.1212)

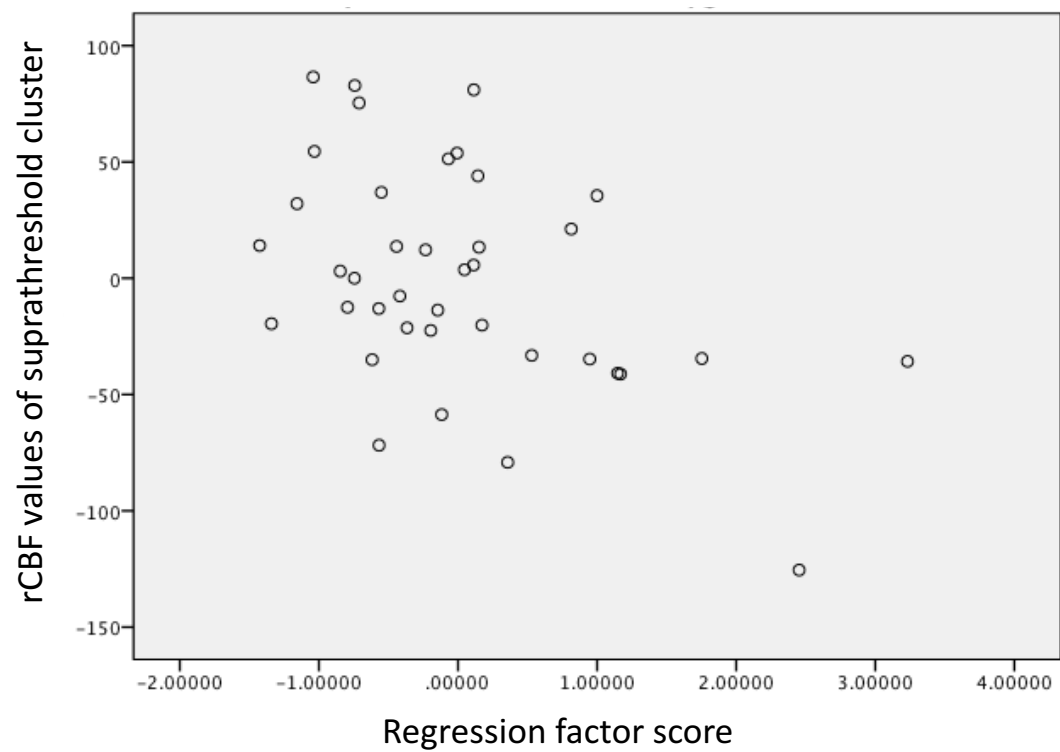

Supplement: Supplementary file 1 — Figure S1 Partial regression plot showing regional blood flow values extracted from the cluster found to be associated with component 1 derived from the principal component analysis. [file EJP-22-1245-s001.pdf]
